# Wu-Chia-Pi Solution Attenuates Carbon Tetrachloride-Induced Hepatic Injury through the Antioxidative Abilities of Its Components Acteoside and Quercetin

**DOI:** 10.3390/molecules171214673

**Published:** 2012-12-11

**Authors:** Steven Kuan-Hua Huan, Kun-Teng Wang, Chia-Jung Lee, Chun-hsien Sung, Ting-Yi Chien, Ching-Chiung Wang

**Affiliations:** 1 Division of Urology, Department of Surgery, Chi Mei Medical Center, No.21, Taikang, Liuying Dist., Tainan 73657, Taiwan; E-Mail: skhsteven@yahoo.com.tw; 2 Graduate Institute of Clinical Medical Research, Taipei Medical University, 250 Wu-Hsing Street, Taipei 11031, Taiwan; 3 School of Pharmacy, College of Pharmacy, Taipei Medical University, 250 Wu-Hsing Street, Taipei 11031, Taiwan; E-Mails: b8706014@tmu.edu.tw (K.-T.W.); m303092003@tmu.edu.tw (C.-J.L.); tim0603kimo@yahoo.com.tw (C.-H.S.); swecon@usc.edu.tw (T.-Y.C.); 4 Section of Biologics & Advanced Therapeutic Product Analysis, Division of Research and Analysis, Food and Drug Administration, No.161-2, Kunyang St, Nangang District, Taipei 11561, Taiwan; 5 Orthopedics Research Center, Taipei Medical University Hospital, Taipei 11031, Taiwan

**Keywords:** Wu-Chia-Pi solution, antioxidative activities, hepatoprotection, carbon tetrachloride, acteoside, quercetin

## Abstract

Wu-Chia-Pi medicated wine, composed nine Chinese medicines soaked in 35% alcohol, is widely used in Asia for its health-promoting functions. However, long-term consumption of alcohol could result in liver dysfunction. In this study, Wu-Chia-Pi solution (WCPS) and extract (WCPE) were prepared by modification of the principals given by the Committee on Chinese Medicine and Pharmacy in Taiwan. The aim of this study was to explore the protective effect of WCPS against carbon tetrachloride (CCl_4_)-induced liver injury and to clarify its active component(s). Antioxidative effects of the test samples were evaluated via MDA inhibition, catalase activity and DPPH-scavenging assays. HPLC was used to analysis the active components. Results showed that WCPS (1 and 5 mL/kg) significantly prevented CCl_4_-induced liver injury without chronic liver toxicity. Referring to the antioxidative activities, WCPE displayed significant MDA inhibitory and DPPH-scavenging activities with IC_50_ values of 0.91 ± 0.03 and 0.60 ± 0.04 mg/mL, respectively. Catalase activity was also enhanced by treatment of WCPE, acteoside and quercetin. Therefore, we suggest that acteoside and quercetin are the major contributors to the antioxidative and hepatoprotective activities of WCPS, and a possible mechanism could be mediated through reduction of oxidative stress.

## 1. Introduction

In traditional Chinese medicine (TCM), people often soak selected herbs in alcohol to obtain medicated wines containing the effective TCM components. Specific Chinese medical herbs are selected make specific medicated wines, the effects of which are usually nourishing and tonic [1]. Wu-Chia-Pi medicated wine, recorded in the Chinese traditional pharmacopoeia as *Tai-Ping-Sheng-Hui-Fang*, is composed of *Acanthopanax gracilistylus* Smith., *Salvia miltiorrhiza* Bunge, *Rehmannia glutinosa* Liboschitz, *Eucommia ulmoides* Oliver, *Zingiber officinale* Roscoe, *Asparagus cochinensis* Merrill, *Lycium chinense* Miller, *Cnidium monnieri* (L.) Cusson and stalactite. The pharmacological effects of Wu-Chia-Pi medicated wine are relief of soreness and weakness of the waist and knees and supplementation of kidney functions [2].

However, it is well-known that chronic alcohol consumption produces major morbidity and mortality worldwide due to alcoholic liver disease (ALD). The impacts of alcohol-induced ALD are systemic, ranging from minimal steatosis to complete liver damage, like fibrosis, cirrhosis, and steato-hepatitis [3]. Chronic alcohol consumption results in fatty liver, which produces several morphological changes and structural lesions in the hepatic lobe. Acute and excessive alcohol consumption can also cause alcoholic pancreatitis and peptic ulcers [4,5]. In addition, about 15% of the morbidity of hepatocellular carcinoma was imputed to alcohol-induced cirrhosis [3]. Reactive oxygen species (ROS) play critical roles in the pathogenesis of ALD. Generation of ROS due to overconsumption of alcohol occurs through the activation of Kupffer cells and oxidation of the liver cytochrome P4502E1 enzyme, eventually causing ALD [6]. Carbon tetrachloride (CCl_4_) is also a well-known toxicant to liver function. Evidence showed that CCl_4_ can also damage hepatocytes through the overproduction of free radicals [7] that can consequently lead to lipid peroxidation, DNA damage, protein denaturation, and cell injury [8,9,10,11]. On the other hand, normal tissues contain biochemical defense systems to scavenge excessive free radicals, including catalase, glutathione peroxidase, and superoxide dismutase [11]. Many antioxidants from natural products possess anti-oxidative abilities. For example, ascorbic acid from citrus fruit and flavonoid compounds from diverse plants are well-known antioxidants.

In order to decrease the alcohol-causing liver injury and maintain the originally pharmacological effects of the active components in the TCM, the alcohol in Wu-Chia-Pi medicated wine was removed by vacuum distillation and the same volume of distilled water added to obtain the Wu-Chia-Pi solution (WCPS) used in this study, whose goal was to evaluate the hepatoprotective effects of WCPS. Besides, the possible protective mechanism and active components of WCPS against CCl_4_-induced hepatic injury were also discussed.

## 2. Results

### 2.1. WCPS Display No Chronic Liver Toxicity in ICR Mice

**Figure 1:**
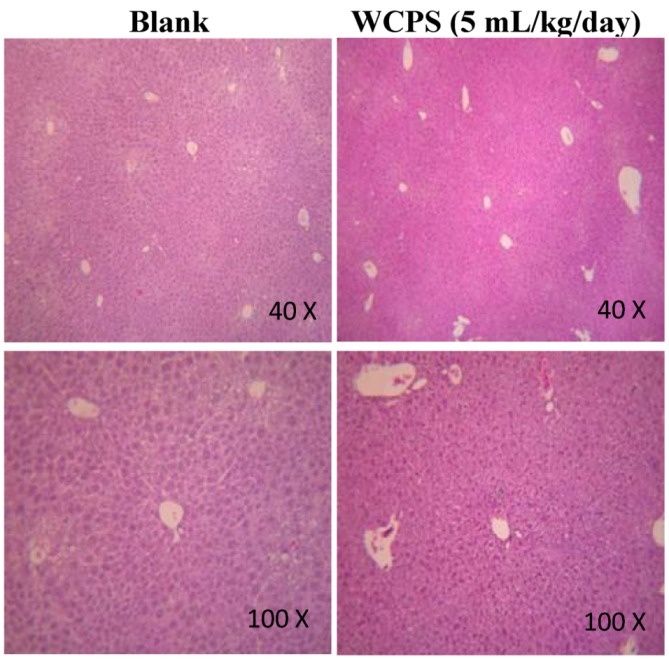
Histological analysis of the chronic liver toxicity of WCPS. Liver tissues were stained with hematoxylin and eosin at 40× and 100× magnifications. The dose of WCPS was 5.0 mL/kg/day.

The composition of WCPS is shown in Table 1. After oral administration of WCPS at the recommended and high dose for 28 days, the GOT and GPT levels were not significantly higher than the blank group (Table 2). This indicated that WCPS had no acute and chronic liver toxicity in ICR mice. In accordance with the pathological examination, the liver architecture was good, and the lobule had a roughly polygonal shape in the low field (Figure 1, Blank group). In a higher field, the hepatic lobule was functionally divided into three zones: zone I encircles the portal tracts where the oxygenated blood from hepatic arteries enters; zone III is located around the central veins, and zone II is located in between. A well-defined lobular structure was observed, and no congestion was seen inside blood vessels in the WCPS group. Therefore, results suggested that WCPS did not cause the liver damage according to the histological analysis.

**Table 1:** Compositions and marker substances in Wu-Chia-Pi medicated wine.

Scientific Name	Ingredients	Used Parts	Marker Substance
*A. gracilistylus* Smith.	38.4 g	Bark	Eleutheroside B_1_
*S. miltiorrhiza* Bunge	38.4 g	Root	Tanshinone IIa
*R. glutinosa* Liboschitz	38.4 g	Root	Acteoside
*E. ulmoides* Oliver	38.4 g	Bark	Quercetin
*Z. officinale* Roscoe	38.4 g	Rhizome	Gingerol
*A. cochinensis* Merrill	38.4 g	Tuber	Asparagine
*L. chinense* Miller	38.4 g	Fruit	Betaine
*C. monnieri* (L.) Cusson	38.4 g	Fruit	Osthol
Stalactite	12.8 g		CaCO_3_
35% alcohol	1 L		

**Table 2:** Chronic liver toxicity of oral administration of the recommended and high dose of WCPS.

	Variations (multiples of day 0)		
	GOT	GPT	GOT/GPT
	Day 28	Day 28	Day 28
Recommended dose ^a^			
Blank	1.05 ± 0.28	0.82 ± 0.23	1.44 ± 0.45
WCPS	0.69 ± 0.27	0.66 ± 0.12	1.03 ± 0.40
High dose ^b^			
Blank	1.09 ± 0.02	1.02 ± 0.07	1.07 ± 0.01
WCPS	0.95 ± 0.08	0.89 ± 0.25	1.07 ± 0.09

^a^ Recommended dose of WCPS was 1.0 mL/kg/day. ^b^ High dose of WCPS was 5.0 mL/kg/day. No significant variations of GOT, GPT and GOT/GPT were found between blank and WCPS in both recommended and high doses. *n* = 5.

### 2.2. WCPS Reduced CCl_4_-Induced Acute Liver Injury in Mice

**Figure 2:**
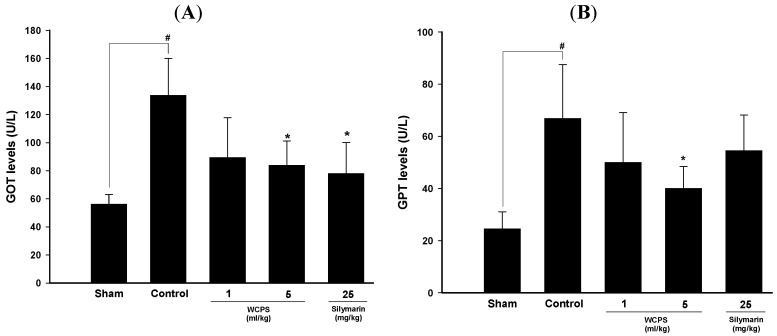
WCPS reduced CCl_4_-induced hepatic injury as evidenced by the lower serum GOT (**A**) and GPT (**B**) levels. ^#^ *p* < 0.05, compard to the sham group. * *p* < 0.05, compared to the control group, *n* = 5. Statistical significant differences were determined by One-Way ANOVA followed by a Fisher LSD *post hoc* test.

To clarify whether WCPS played a hepatoprotective role, we used a CCl_4_-induced acute liver damage model. As shown in Figure 2, GOT and GPT levels of the control group were 2-fold higher than those of the sham group, indicating that CCl_4_ significantly damaged hepatocytes and resulted in the release of GOT and GPT into serum. However, WCPS significantly reduced serum GOT (Figure 2A) and GPT (Figure 2B) levels in a dose-dependent manner, while a high dosage of WCPS displayed the same protective effects as silymarin, our positive control.

### 2.3. WCPE Displayed Significant Anti-oxidative Abilities

**Figure 3:**
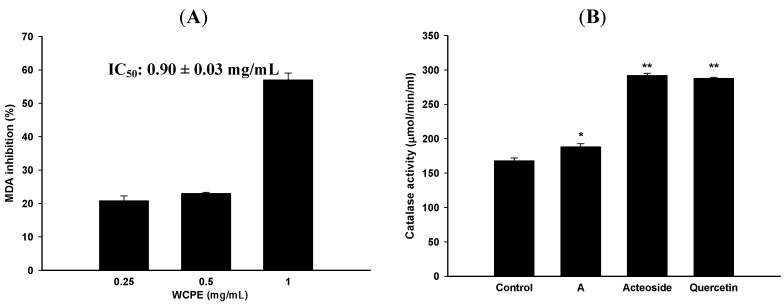
MDA inhibitory effects (**A**) and catalsed activity (**B**) of WCPE and marker substances. A, WCPE; Control, untreated group. Concentrations of WCPE and marker substances in catalase assay were 10 mg/mL and 5 μM, respectively. * *p* < 0.05, ** *p* < 0.005, compared to the control group. Statistical significant differences were determined by One-Way ANOVA followed by a Fisher LSD *post hoc* test.

According to etiological studies, alcohol and CCl_4_ can induce oxidative stress and cause hepatic diseases. Thus, the MDA inhibitory effect of WCPE was firstly evaluated using liver homogenates. As shown in Figure 3A, WCPE (1 mg/mL) displayed MDA inhibitory effects with a 50% inhibitory concentration (IC_50_) value of 0.90 ± 0.03 mg/mL. Moreover, catalase acted as an internal anti-oxidative enzyme. WCPE significantly upregulated the catalase activity in liver tissue homogenates (Figure 3B). Results indicated that WCPE could inhibit lipid peroxidation and enhance catalase activity in the liver. WCPE (1 mg/mL) also displayed significant DPPH-scavenging effects, while the IC_50_ value was 0.60 ± 0.04 mg/mL (Table 3).

**Table 3:** DPPH scavenging effects of WCPE and its marker substances.

	DPPH scavenging effects		
Sample	Inhibition ^a^ (%)	IC_50_ value ^c^	
WCPE	72.75 ± 2.08	0.60 ± 0.04	mg/mL
Acteoside	77.23 ± 0.16	17.12 ± 0.30	μM
Quercetin	69.31 ± 0.32	23.02 ± 0.57	μM
Osthol	0.09 ± 0.16	-	
Betaine	0.36 ± 0.63	-	
Tanshinone IIa	39.44 ± 0.16	-	
α-Tocopherol ^b^	64.30 ± 1.92	50.39 ± 3.62	μM

^a^ Concentrations of WCPE and marker substances were 1 mg/mL and 125 μM, respectively; ^b^ α-Tocopherol was the positive control; ^c^ IC_50_, 50% inhibitory concentration.

### 2.4. Acteoside and Quercetin Were the Major Anti-Oxidative Components in WCPS

**Figure 4:**
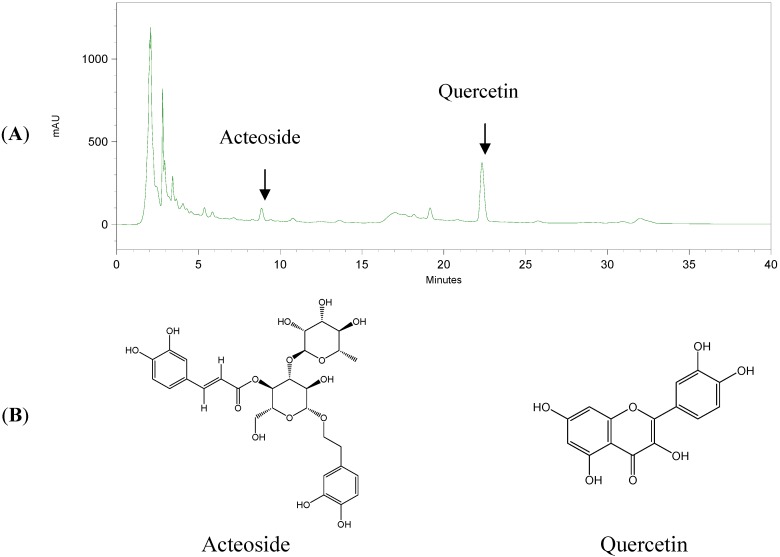
The HPLC fingerprint of WCPE (**A**) and the chemical structures of acteoside and quercetin (**B**). Retention times of acteoside and quercetin were 8.9 and 22.5 min, respectively. The horizontal axis represented time in minutes, and vertical axis represented mAU, the intensity of the UV absorbance values.

To further clarify the active components of the anti-oxidative abilities in WCPS, we used a DPPH-scavenging assay. As shown in Table 3, acteoside and quercetin displayed stronger DPPH-scavenging effects than the other marker substances, with respective IC_50_ values of 17.12 ± 0.30 and 23.02 ± 0.57 μM. In terms of the catalase activity assay, acteoside and quercetin at 5 μM also significantly increased the liver catalase activity (Figure 3B). According to the above results, acteoside and quercetin displayed the most significantly anti-oxidative effects and were used as bioactive marker substances of WCPS. Bioactive marker substances in WCPS and WCPE were detected by HPLC analysis. Figure 4 shows the fingerprint of WCPE, and the respective retention times of acteoside and quercetin were 9.1 and 22.5 min. In addition, the concentrations of these two compounds were analyzed. As shown in Table 4, contents of acteoside in WCPS and WCPE were 23.51 ± 3.67 μg/mL and 0.36 ± 0.01 μg/mg, respectively. Contents of quercetin in WCPS and WCPE were 97.95 ± 2.29 μg/mL and 3.03 ± 0.07 μg/mg, respectively.

**Table 4:** Contents of acteoside and quercetin in WCPS and WCPE.

Sample	Content	
	Acteoside	Quercetin
WCPS (μg/mL)	23.51 ± 3.67	97.95 ± 2.29
WCPE (μg/mg)	0.36 ± 0.01	3.03 ± 0.07

## 3. Discussion

In TCM, medicated wine is used for body-strengthening functions. Wu-Chia-Pi medicated wine, a commonly used medicated wine in Taiwan, is usually used to supplement kidney functions, enhance blood circulation, and relieve pain [12]. The recommended dosage of Wu-Chia-Pi medicated wine consumption in Taiwan is 1.0 mL/kg/day. However, evidence shows that the chronic consumption of alcohol can cause liver and kidney damage [13,14]. One of the alcohol-induced health-damaging factors is ROS attack. Uncontrolled ROS rapidly cause the denaturation of membrane lipids and proteins, and damage and mutations to DNA. In addition, it is believed that the overproduction of ROS is related to many metabolic and inflammatory diseases, including dyslipidemia and fatty liver [15].

Nowadays, Chinese herbs are popularly used for health-promoting functions. Marker substances in the crude extracts can alleviate and prevent ROS-induced diseases [16]. In this study, we removed the alcohol from the Wu-Chia-Pi medicated wine and added the same volume of distilled water to obtain the WCPS, which was freeze-dried to obtain the WCPE. WCPS was used to determine the protective effects against CCl_4_-induced hepatic injury. Before performing the liver protection assay, it should be confirmed whether the WCPS had liver toxicity. The chronic liver toxicity study was designed and conducted to determine the toxicity profile of WCPS after daily administered for 28 days in ICR mice. GOT and GPT, two specific metabolic transaminases in hepatocytes, were used to assay the liver functions in WCPS-treated ICR mice. In Cohen’s study, differences in the clinical meanings of GOT and GPT levels were clarified. Patients with fatty liver and hepatitis had higher GOT levels, while higher GPT levels were usually found in hepatic steatosis patients [17,18]. As summarized in Table 2, GOT and GPT levels were elevated by consumption of the recommended and high dosage of WCPS. After consumption of WCPS for 28 days, both GOT and GPT were the same as the blank group, suggesting that no liver toxicity was found. To further evaluate the hepatoprotective effects of WCPS, CCl_4_-induced hepatic injury model was used. CCl_4_ is a well-known chemical which causes free radical-mediated liver injury. The CCl_4_-reactive free radicals, such as trichloromethyl radical (CCl_3_^•^) and trichloromethyl peroxy radical (CCl_3_ OO^•^), produced by cytochrome P450 of liver microsomes were the major reactive intermediates causing liver injuries. Evidence has showed that trichloromethyl radical and trichloromethyl peroxy radical could react with cell membrane disintegration of hepatocytes, resulting in release of the GOT and GPT [19]. In this study, WCPS significantly and dose-dependently decreased CCl_4_-induced hepatic injury, suggesting the strong anti-oxidative activities of WCPS (Figure 2). In addition, the hepatoprotective effects of WCPS (5 mL/kg) were the same as our positive control, silymarin.

Since the WCPS displayed the obviously hepatoprotective effects, the possible protective mechanism was discussed. As the previous described, ROS attack is the major reason for CCl_4_-induced hepatic injury. Different antioxidative assays were employed to evaluate the free radical-scavenging activities of WCPS. We used WCPE instead of WCPS to evaluate the antioxidative activities because WCPE was more convenient than WCPS in calculating the IC_50_. Firstly, WCPE displayed obvious antioxidative activities in MDA inhibitory and DPPH-scavenging assays, with IC_50_ values of 0.90 ± 0.03 and 0.60 ± 0.04 mg/mL, respectively. Through the above findings, we knew that WCPE had potential antioxidative activities and wanted to find out its active components. Among the five different marker substances in WCPE, acteoside from *R. glutinosa* and quercetin from *E. ulmoides* displayed the most significant DPPH-scavenging and catalase-enhancing activities (Table 3 and Figure 3B). Acteoside is classified as a phenylethanoid glycoside which is widely distributed in tonic TCMs (Figure 4A). The pharmacological effects of acteoside, including its anti-oxidation, skin-whitening, anti-inflammatory, and neuroprotective properties, are well-studied. Evidence showed that acteoside displays protective effects against CCl_4_- and D-galactosamine-induced liver injury, while the possible mechanism was also through free radical-scavenging effects [20,21,22]. Quercetin is also a well-known plant-derived flavonoid (Figure 4B) and is well-known for its anti-oxidative and anti-inflammatory activities. In previous studies, quercetin prevented ethanol-induced hepatic injury through its anti-oxidative abilities in both *in vitro* and *in vivo* studies [23,24,25]. According to the previous information, we assumed that acteoside and quercetin contributed the most significant antioxidative and hepatoprotective activities of WCPS, even though the traditional Chinese pharmacopeia did not mention its hepatoprotective ability. Taken together, WCPS displayed significant hepatoprotective effects, while acteoside and quercetin were the active marker substances. However, the protective mechanism of WCPS was through scavenging external ROS and increasing internal catalase activities.

## 4. Experimental

### 4.1. Materials

Traditional Chinese herbs, *Acanthopanax gracilistylus* (AG-001), *Salvia miltiorrhiza* (SM-001), *Rehmannia glutinosa* (RG-001), *Eucommia ulmoides* (EU-002), *Zingiber officinale* (ZO-002), *Asparagus cochinensis* (AC-001), *Lycium chinense* (LC-002), *Cnidium monnieri* (CM-002), and stalactite (CaCO_3_, S-001), were collected from Chinese herbal pharmacies in Taipei, Taiwan, in November 2008. The eight TCMs were identified by Prof. Chang Hsien-Chang from the Taiwan Food and Drug Administration. A voucher specimen of each material was deposited in the Graduate Institute of Pharmacognosy Science, Taipei Medical University. Carbon tetrachloride, soybean oil, *tert*-butylhydroperoxide, thiobarbituric acid, 1,1-diphenyl-2-picrylhydrazyl (DPPH) and silymarin were purchased from Sigma-Aldrich (St. Louis, MO, USA). HPLC grade acetonitrile was bought from Merck (Darmstadt, Germany). Silymarin was dissolved in phosphate-buffered saline, as a stock solution and kept at 4 °C.

### 4.2. Animals

ICR male mice weighing 25 ± 2 g were obtained from BioLASCO Taiwan (Yilan, Taiwan) and kept on a 12:12-h day-night cycle. Animals were maintained at 21 ± 2 °C and provided food and water *ad libitum*. All experimental procedures followed ethical regulations of Taipei Medical University (approval no. LAC-97-0122).

### 4.3. Preparation of Wu-Chia-Pi Solution (WCPS) and Extract (WCPE)

WCPS preparation protocol was modified from the principals of Wu-Chia-Pi medicated wine as determined by the Committee on Chinese Medicine and Pharmacy (CCMP) in Taiwan [12]. All materials as Table 1 were soaked in 35% alcohol for 30 days. WCPS was prepared by removing the alcohol by vacuum distillation and adding the same volume of distilled water (1 L). In addition, the WCPS was freeze-dried to obtain the WCPE and the yield was 6.56%.

### 4.4. Hepatoprotective Effects of WCPS

#### 4.4.1. Chronic Liver Toxicity in ICR Mice

We divided ICR mice into two groups, including a blank group (which was fed deionized water) and a WCPS group. The intake dosage was modified from the CCMP regulations. In this study, ICR mice were orally administrated the recommended dosage (1 mL/kg/day) and a high dosage (5 mL/kg/day) for 28 days. On days 0 and 28, serum was obtained for glutamic oxaloacetic transaminase (GOT) and glutamic pyruvic transaminase (GPT) assay with a Fuji DRI-CHEM 3500i analyzer (Tokyo, Japan). At the end of 4 weeks of treatment, ICR mice were sacrificed, and the liver tissue was fixed for a histopathological analysis.

#### 4.4.2. CCl_4_-Induced Hepatic Injury in ICR Mice

The assay protocol of CCl_4_-induced hepatic injury was modified from that of a previous study [26]. The intake dosage was modified from the CCMP regulations. ICR mice were given a recommended dosage (1 mL/kg/day), a high dosage (5 mL/kg/day) of WCPS and silymarin (25 mg/kg, 0.1 mL/10 g mice) every day for 7 days. On day 7, mice were given WCPS 1 h before an intraperitoneal (*i.p.*) injection of a 0.4% CCl_4_-soybean oil solution. Control mice were given distilled water and the same dose of an *i.p.* CCl_4_-soybean oil injection. After 6 h, the mice were sacrificed and whole blood was collected via heart puncture. Whole blood was centrifuged at 1,500 rpm for 10 min at 4 °C to obtain the serum.

### 4.5. Antioxidative Assays

#### 4.5.1. Lipid Peroxidation Assay

The protocol of the MDA inhibitory effects was modified from our previous study [27]. Normal ICR mice liver tissue was homogenized in phosphate-buffered saline (PBS). Homogenized tissues were quantified and treated with 125 mM *tert*-butylhydroperoxide (TBH) with or without WCPE, and eventually reacted with thiobarbituric acid (TBA) to form the pink adduct, MDA. The optical density was measured at 530 nm with a μQuant spectrophotometer (BioTek, Winooski, VT, USA).

#### 4.5.2. DPPH Radical-Scavenging Assay

Assay protocol of DPPH-scavenging activities was described according to a previous study [26]. Different dosages of WCPE and marker substances of each plant material (acteoside, quercetin, osthol, betaine, and tanshinone IIa from *R. glutinosa*, *E. ulmoides*, *C. monnieri*, *L. chinense*, and *S. miltiorrhiza*, respectively) were mixed with DPPH solution (100 μM) in a 96-well microplate at room temperature for 10 min. α-Tocopherol was used as the positive control. DPPH level of each well was evaluated by detecting the optical density of each well at 530 nm. Inhibition index (%) was calculated according to the following equation: I% = [1 − (T/C)] × 100%, where T and C represent the mean optical density of the treated group and vehicle control group, respectively. In accordance with the I% of the dose-response curve (WCPE: 31.25, 62.5, 125, 250, 500 and 1,000 μg/mL; test compounds: 3.91, 7.8, 15.625, 31.25, 62.5 and 125 μM), the concentration of the tested compound giving 50% of DPPH free radical inhibition (IC_50_ value) was estimated.

#### 4.5.3. Catalase Assay

The catalase assay protocol was modified from that of a previous study [28]. Ferrous oxidation in a xylenol orange solution was used as a discontinuous method to measure catalase activity. Catalase activity was represented in units of μmol/min/mL through detection at 560 nm.

### 4.6. High-Performance Liquid Chromatographic Analysis of Marker Substances in WCPS and WCPE

The HPLC apparatus was composed of an SCL-10Avp System Controller, an LC-10ATvp Liquid Chromatograph Pump, an SPD-M10A Diode Array Detector, an SIL-10Avp Auto Injector, a CTO-10A Column Oven, FCV-10Avp Flow-Channel Selection Valves (Shimadzu, Tokyo, Japan), and an ERC-3415 Degasser (ERC, Altegolfsheim, Regensburg, Germany). The stationary phase consisted of a Purospher^®^ STAR RP-18e reversed-phase column (5 μm, 4 mm i.d. × 250 mm, Merck). The mobile phase system was 0–10 min of CH_3_CN:H_2_O of 18:82 and 10–40 min of CH_3_CN:H_2_O of 23:77. The flow rate was 1 mL/min, and the oven temperature was maintained at 40 °C. We used a UV wavelength of 280 nm to detect the contents of acteoside and quercetin in WCPS and WCPE. WCPE was dissolved in MeOH to a concentration of 150 mg/mL. The sample solutions (WCPS and WCPE) were then filtered through a 0.45-μm syringe filter, and a 10-μL volume was directly injected into the HPLC system.

Acteoside and quercetin were accurately weighed and dissolved in methanol to give serial concentrations in the ranges of 12.5–200 μg/mL. Calibration curves were plotted after linear regressions of the peak areas. Calibration equations for acteoside and quercetin were y = 7408.7x + 582330 (R^2^ = 0.999) and y = 12345x + 756603 (R^2^ = 0.999), respectively. The retention times of the bio-marker substances were 8.9 and 22.5 min for acteoside and quercetin, respectively (Figure 4). The linearity of the peak area (y) *vs*. concentration (x, μg/mL) curve for acteoside and quercetin was used to calculate the contents of the biomarker substances in WCPS and WCPE.

### 4.7. Statistical Analysis

Data are presented as the mean and standard deviation (SD) and were analyzed with One-Way ANOVA followed by a Fisher LSD *post hoc* test using the SPSS 17 software (SPSS Inc., Chicago, IL, USA).

## 5. Conclusions

In summary, we found that the WCPS displayed significant hepatoprotective effects against CCl_4_-induced hepatic injury. The possible mechanism was the anti-oxidative activities of WCPS, and the major active components were acteoside and quercetin.
